# P-446. Clinical Characteristics and Outcomes of Pediatric Patients with Gram-negative Bacteremia at a Large Academic Medical Center

**DOI:** 10.1093/ofid/ofaf695.661

**Published:** 2026-01-11

**Authors:** Katie B Olney, David S Burgess, Donna R Burgess

**Affiliations:** University of Kentucky HealthCare, Lexington, KY; University of Kentucky, Lexington, KY; UK HealthCare, Lexington, KY

## Abstract

**Background:**

Gram-negative bacteremia (GNB) in pediatric patients presents a serious clinical challenge, yet comprehensive data on its epidemiology, management, and outcomes in this population are limited. This study aimed to characterize the clinical features, microbiologic profile, and factors associated with mortality in pediatric patients with monomicrobial GNB at a large academic medical center.

**Methods:**

A retrospective cohort study was conducted among 970 patients with GNB identified from institutional microbiology records; 62 pediatric patients (< 18 yrs) with monomicrobial GNB were included. Demographics, clinical characteristics, microbiology, time to diagnostics and interventions, and outcomes were analyzed.

**Results:**

Of the 62 pediatric patients, 64.5% of infections were community-acquired. The cohort was predominantly White (75.8%) followed by 9.7% Black, 9.7% Hispanic, and 4.8% other. The most common pathogens were E. coli (51.6%) and Klebsiella spp. (12.9%). Overall mortality was 11.3%, with a 90-day readmission rate of 25.8%. ICU admission occurred in 19.4% of cases. ID consultation was obtained in 58.1% of cases, with a median (IQR) time to consult of 22.2 hrs (6, 43.9). Median hospital LOS was 15.5 days (10, 72); ICU LOS was 10.4 days (6.4, 18.1). Time to ePlex® result and susceptibility data were 15.6 hrs (13.1, 23.5) and 3.6 days (2.6, 5.7), respectively. Median duration of antimicrobial therapy was 10 days (6.9, 13.5). The Charlson Comorbidity Index (CCI) was low in 79%, moderate in 12.9%, and high in 8.1%, with higher CCI significantly associated with mortality (p=0.003). Mortality was highest among patients with P. aeruginosa (100%, N=3) and E. coli (9.4%, N=3) bacteremia. No deaths occurred among patients with Klebsiella spp. or S. marcescens infections.

**Conclusion:**

At our institution, pediatric Gram-negative bacteremia was predominantly community-acquired and most frequently caused by E. coli. Although overall mortality was low, it was notably higher in patients with P. aeruginosa infections and elevated CCI scores. Ongoing efforts are needed to enhance risk stratification and optimize management strategies in this high-risk population.

**Disclosures:**

All Authors: No reported disclosures

